# The Synergistic Repressive Effect of NF-κB and JNK Inhibitor on the Clonogenic Capacity of Jurkat Leukemia Cells

**DOI:** 10.1371/journal.pone.0115490

**Published:** 2014-12-19

**Authors:** Xinli Liu, Jun Zhang, Jing Li, Andrew Volk, Peter Breslin, Jiwang Zhang, Zhou Zhang

**Affiliations:** 1 Department of Biology, College of Life and Environment Science, Shanghai Normal University, Shanghai, People's Republic of China; 2 College of Life Science and Biopharmacy, Shenyang Pharmaceutical University, Shenyang, People's Republic of China; 3 Oncology Institute, Cardinal Bernardin Cancer Center and Departments of Pathology and Molecular/Cellular Physiology, Loyola University Chicago, Maywood, IL 60153, United States of America; Columbia University, United States of America

## Abstract

Deregulation of Nuclear Transcription Factor-κB (NF-κB) and Jun N-terminal kinase (JNK) signaling is commonly detected in leukemia, suggesting an important role for these two signaling pathways in the pathogenesis of leukemia. In this study, using Jurkat cells, an acute T-lymphoblastic leukemia (T-ALL) cell line, we evaluated the effects of an NF-κB inhibitor and a JNK inhibitor individually and in combination on the proliferation, survival and clonogenic capacity of leukemic cells. We found that leukemic stem/progenitor cells (LSPCs) were more sensitive to NF-κB inhibitor treatment than were healthy hematopoietic stem/progenitor cells (HSPCs), as shown by a reduction in the clonogenic capacity of the former. Inactivation of NF-κB leads to the activation of JNK signaling in both leukemic cells and healthy HSPCs. Interestingly, JNK inhibitor treatment enhanced the repressive effects of NF-κB inhibitor on LSPCs but prevented such repression in HSPCs. Our data suggest that JNK signaling stimulates proliferation/survival in LSPCs but is a death signal in HSPCs. The combination of NF-κB inhibitor and JNK inhibitor might provide a better treatment for T-ALL leukemia by synergistically killing LSPCs while simultaneously preventing the death of normal HPCs.

## Introduction

Acute T-lymphoblastic leukemia (T-ALL) is a malignant hematopoietic disorder characterized by uncontrolled proliferation and infiltration of immature T lymphoblasts into hematopoietic tissue such as bone marrow, spleen and peripheral blood, as well as other organs [1]. Intensive chemotherapy is still the only treatment for this fatal disease. But such treatment typically kills tumor cells and normal tissue cells indiscriminately [2]–[5]. As a consequence, the quality of life as well as the prognosis for T-ALL patients is still very poor. More specifically targeted and less toxic forms of therapy are critically needed to improve the outcome for these patients.

Nuclear Transcription Factor-κB (NF-κB) is a family of transcription factors that regulate the expression target genes, and have been associated with diverse biological and pathological processes [6], [7]. In most types of cells, NF-κB is normally inactivated by members of the IκB family of inhibitory proteins which sequester NF-κB within the cytoplasm and subject NF-κB to proteasome-mediated degradation. Extracellular stimuli such as TNF-α and IL-1 induce the phosphorylation and activation of IκB kinases (IKKs). Activated IKKs phosphorylate IκB proteins, releasing NF-κB from inhibition by the former [6]–[8]. This allows the translocation of NF-κB from the cytoplasm into the nucleus, where it regulates the expression of genes responsive to it. Hundreds of NF-κB target genes have been identified; their protein products are involved in diverse key cellular and organismal processes, including cell proliferation, cell survival, the cellular stress response, innate immunity and inflammation [9].

Deregulation of the NF-κB signaling is commonly detected in many human cancers, including leukemia. It was demonstrated that NF-κB is an important mediator of immune and inflammatory reactions. In most inflammation-related cancers, the abnormal activation of NF-κB and the chronic inflammatory reaction induced by inflammatory cytokines produced by tumor cells and/or tumor environmental cells [10]. Hence, inhibition of NF-κB signaling has been proposed to be a potential therapeutic option in the treatment of cancer, especially for such inflammation-related cancers. In T-ALL, in addition to inflammatory cytokine stimulation, abnormal activation of T-cell receptor signaling, constitutive activation of Notch signaling and the inactivation of Pten signaling, all due to mutations in key components of these signal pathways, are major causes leading to the activation of NF-κB signaling in tumor cells [11], [12]. The NF-κB signaling pathway is highly activated in leukemic cells isolated from a majority of T-ALL patients [13]. Elevated NF-κB activity in leukemic cells provides a survival signal in these cells by up-regulating anti-apoptotic genes; such might be a major causal factor for the drug resistance often observed in such cancer cells. Thus, NF-κB signaling is also a critical target for anti-T-ALL therapy, especially for patients with drug-resistant tumors. NF-κB inhibitors have been tested in the clinical setting to treat T-ALL patients [14], [15] but with only limited success. The clinical use of NF-κB inhibitors in cancer therapy is limited by their side-effects and attenuated killing effects on tumor cells *in vivo*. The potential side-effects of NF-κB inhibitors include compromised T/B-cell immunity, inflammatory tissue damage, and an increased risk for the development of skin and liver cancer [16]–[18]. Interestingly, both the side-effects and reduced anti-tumor effects of NF-κB inhibitors *in vivo* might be due to the excessive sensitivity of NF-κB-inactivated cells to TNF-induced activation of JNK signaling [19].

Accumulating evidence suggests that TNF can stimulate both survival and death signals within the same type of cells in a context-dependent fashion. TNF-dependent survival signals are primarily mediated by the canonical NF-κB pathway, while the TNF-induced death signal is driven by caspase-8-dependent apoptosis or RIP1/3-dependent necroptosis [20]–[23]. In addition, TNF also stimulates the activation of Jun N-terminal kinase (JNK) signaling [24]. JNK is a member of the mitogen activated protein kinase (MAPK) family. Interestingly, it has been demonstrated that JNK signaling also induces the dual roles of survival and death in a cell context-dependent fashion [25], [26]. JNK signaling induces cell death through phosphorylation/inactivation of Bcl2-related anti-apoptotic signaling and promotes cell proliferation/survival through AP-1 (c-Jun/Fos) complex-regulated gene expression [26]. Thus, we predict that inhibition of JNK signaling might provide improved anti-cancer therapy when combined with NF-κB inhibition, potentially synergistically killing cancer cells while protecting normal tissue cells from TNF-induced damage. To test this hypothesis, in the present study, we compared the response of Jurkat T-ALL cells and HSPCs to JNK inhibitor and NF-κB inhibitor treatment both individually and also in combination. We demonstrated that the inhibition of NF-κB induces the activation of JNK signaling in both Jurkat cells and HSPCs. JNK stimulates a survival/proliferation signal in Jurkat cells whereas it promotes death signaling in HSPCs. Our data indicate that the inhibition of JNK signaling not only significantly sensitizes T-ALL cells to NF-κB inhibitor treatment, but also protects HSPCs from the toxic effects of such treatment. Hence, the simultaneous use of an NF-κB inhibitor together with a JNK inhibitor may synergize to provide a promising novel strategy for therapeutic intervention in T-ALL.

## Materials and Methods

### Reagents

Recombinant human-IL-3 (rhIL-3), rhIL-6, rhSCF were purchased from eBioscience (San Diego). TNF-α was purchased from BD Biosciences. BAY11-7082 (NF-κB inhibitor) was purchased from Beyotime Institute of Biotechnology. SP600125 (JNK inhibitor) was purchased from Cell Signaling. Cell Lysis Buffer (10×) was obtained from Cell Signaling and supplemented with proteinase inhibitors and phosphatase inhibitors (Roche Diagnostics). Anti-β-actin antibody was obtained from Santa Cruz Biotechnology. Anti-p65, p-p65, JNK, p-JNK, Caspase-3, Caspase-8 and Caspase-9 primary as well as requisite secondary antibodies were obtained from Cell Signaling. Annexin-V-FITC/PI Apoptosis Detection Kit was purchased from BD Biosciences.

### Cell culture

Jurkat cells, a human T-ALL cell line, were obtained from ATCC and incubated in 10% FBS RPMI-1640 medium at 37°C, 100% humidity and 5% CO_2_. Peripheral blood CD34^+^ HSPCs used in this study are residual samples which were collected from healthy donors for stem cell transplantation. These cells were stored as frozen tissue in the tissue bank at the Cardinal Bernardin Cancer Center, Loyola University Chicago. IRB protocol #205719090113 allows permission for the use of these samples, as approved by the IRB committee of Loyola University Chicago. They were cultured in RPMI-1640 medium supplemented with 10% FBS, penicillin/streptomycin, 100 ng/ml rhSCF, 10 ng/ml rhIL-6, and 10 ng/ml rhIL-3.

### Methyl thiazolyl tetrazolium (MTT) assay

Exponentially-growing Jurkat cells or CD34^+^ HSPCs were seeded into 96-well flat-bottomed plates in triplicate at a cell density of 1×10^5^ cells per ml. of medium. NF-κB inhibitor BAY11-7082 and JNK inhibitor SP600125 were dissolved in DMSO and added to the each well at indicated concentrations. An equivalent volume of DMSO was used as vehicle control in all of our experiments. Forty-four hours after inhibitor treatment, 5 mg/ml of MTT was added to each well and further incubated for 4 hours in order to allow MTT to be reduced by enzymes from metabolically active cells. Subsequently, 100 µl of 10% SDS was added to each well to dissolve the formazan for 6 hours. Spectrophotometric absorbance in each well was recorded at 570 nm using a *SpectraMax* plate reader (Epoch Biotek technology). The absorbance reflects the number of viable cells. Triplicate experiments were performed in all our studies. All data were verified by three individual experiments.

### Colony-Forming Unit (CFU) Assay

Exponentially-growing Jurkat cells or CD34^+^ HSPCs were incubated in the indicated medium at a cell density of 1×10^5^ cells/ml with or without inhibitors for 24 hours. All cells were isolated from the plate, centrifuged, and resuspended into 1 ml of supplemented medium; this process was repeated. Jurkat cells or CD34^+^ HSPCs were seeded into *MethoCult* GF M3434 (StemCell) at 1000 cells/ml, incubated at 37°C, 100% humidity and 5% CO_2_ for 7 days (Jurkat cells) or 10 days (CD34^+^ HSPCs). Numbers of colonies were counted according to the manufacturer's instructions.

### Retroviral transduction

Retroviral production and infection were performed as reported [27]. MIGR1 (Cat. 27490) and pMIEG3-JunDN (Cat. 40350) retroviral plasmids were obtained through Addgene. MIGR1-DN-AP1 plasmid was generated by subcloning the JunDN fragment from pMIEG3-JunDN plasmid into the multiple clone site of MIGR1 plasmid. High titer of DN-AP1 or empty vector (Migr1) expressing retrovirus were produced by co-transfecting Phoenix cells with a retroviral vector containing DN-AP1 or vector-only together with packaging vectors using the Calphos Mammalian Transfection kit (Takara Bio Inc.). Retroviral supernatants were harvested 24 and 48 h. after transfection. Jurkat cells were transduced with such virus by spinoculation at 32°C, 2000 rpm for 4 h. Transduced cells (GFP+) were purified by FACS and treated with the indicated reagents for CFU assay.

### Western blot analysis

Western blot analysis was performed using standard techniques. Briefly, cell lysates from Jurkat cells and CD34^+^ HPSCs were incubated with the indicated concentrations of BAY11-7082, SP600125 and TNF for 2 hours. Protein lysates were run on a precast 10% gradient polyacrylamide gel to detect the levels of p-NF-κB and p-JNK. Total NF-κB, JNK and β-actin were used as protein loading controls. The activities of Caspases 3, 8 and 9 were evaluated by detecting levels of the cleaved forms of Caspases 3, 8 and 9. β -actin and full-length Caspases 3, 8 and 9 were used as protein loading controls.

### Wright-Giemsa staining

Exponentially-growing Jurkat cells or CD34^+^ HSPCs were incubated in medium with or without inhibitors at a cell density of 1×10^5^ cells/ml. for 48 hours. All cells were isolated from the plate, centrifuged, and resuspended into 1 ml. of supplemented medium. The cells were spread on clean glass slides until the slides were dry. Wright-Giemsa stain was added to the smear in sufficient quantity to cover the entire surface, and incubated at room temperature for 3–4 minutes, followed by the addition of 2.0 ml of PBS, pH 6.5 for another 6 to 8 minutes. Stained smears were rinsed with water until the edges showed a faint pinkish-red color. Cells were observed and photographed using oil immersion microscopy.

### Annexin V–FITC/PI staining

Exponentially-growing Jurkat cells or CD34^+^ HSPCs were incubated in medium with or without inhibitors at a cell density of 1×10^5^ cells/ml for 48 hours. Cells were then stained with FITC-conjugated Annexin-V, followed by propridium iodide (PI) staining in Annexin binding buffer following the instructions provided by the manufacturer (BD Biosciences). The number of apoptotic cells was analyzed by flow cytometry for percentages of Annexin V^−^/PI^+^ and Annexin V^+^/PI^+^ populations.

### Statistical analysis

Statistical analysis was performed using *GraphPad Prism* 5.0 software (GraphPad, San Diego, CA, USA). Results shown are expressed as mean ± standard deviation. Differences between 2 groups were examined for statistical significance using the Student's t-test. When more than two groups were compared, analysis of Variance (ANOVA) is used to determine statistical significance.

## Results

### Jurkat cells are more sensitive to NF-κB inhibitor treatment than are normal HSPCs

**Figure 1:**
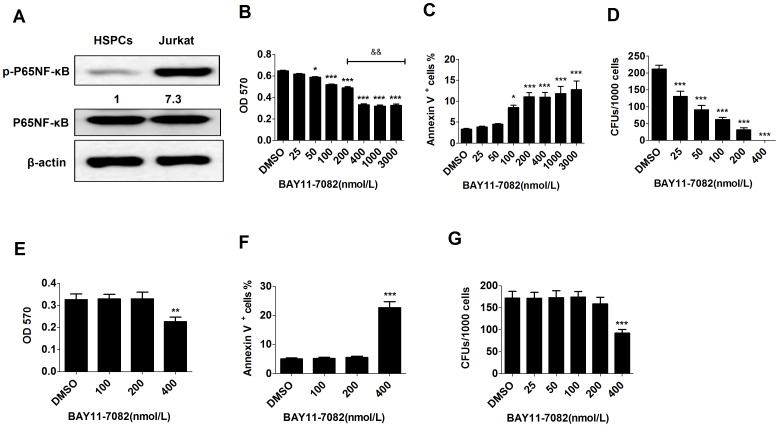
The effect of NF-κB inhibitor on viability and CFU capacity of Jurkat cells and normal HSPCs. A. Cell lysates were collected from CD34^+^ HSPCs and Jurkat cells. NF-κB activity was examined by Western blotting to detect p-p65 NF-κB levels. B–D: B. Jurkat cells were treated with increasing concentrations of BAY11-7082. Cells were collected after 48 hours of treatment. Cell growth was examined by MTT assay; C. Cell viability was determined by Annexin-V staining and flow cytometric analysis; D. Clonogenic capacity was evaluated by CFU assay. E–G: E. CD34^+^ normal HPSCs were treated with increasing concentrations of BAY11-7082. Cells were collected after 48 hours of treatment. Cell growth was examined by MTT assay; F. Cell viability was determined by Annexin-V staining and flow cytometric analysis; G. Clonogenic capacity was evaluated by CFU assay. Data were collected from triplicate experiments and repeated for three times. Data shown in Figures are an average of three independent experiments. *P<0.05; **P<0.01; ***P<0.001 compared to DMSO-treated group.

Compared to normal CD34^+^ HSPCs, Jurkat T-ALL cells have higher NF-κB activity as shown by higher levels of p-NF-κB expression (**Fig. 1A**). To study the role of NF-κB on the growth and survival of T-ALL cells, Jurkat cells were treated with increasing concentrations of BAY11-7082 for 48 hours. Cell growth and death were assessed by MTT assay and Annexin-V staining, respectively. MTT analysis revealed that treatment of Jurkat cells with 100 nmol/L BAY11-7082 resulted in a slight but statistically significant decrease in absorbance (0.52±0.006 *vs*. 0.65±0.006 in vehicle control group). A marked decrease was observed at a concentration of 400 nmol/L (0.33±0.01 *vs*. 0.65±0.006 in vehicle control group) (**Fig. 1B**). Interestingly, increased BAY11-7082 concentration (above 400 nmol/L) did not lead to an additional MTT reduction, suggesting BAY11-7082 might only repress the growth of a portion of leukemic cells. Consistently, as shown by Annexin-V staining, a substantial degree of cell death was observed when cells were exposed to 100–400 nmol/L BAY11-7082 compared to the vehicle control group. However, death of treated cells was not further increased at BAY11-7082 concentrations >400 nmol/L. (**Fig. 1C**). CFU assay demonstrated a very significant dose-dependent response of Jurkat cells to BAY11-7082. In this assay, Jurkat cells were first treated with increasing dosages of BAY11-7082 for 24 hours, then seeded for CFU. As shown in **Fig. 1D**, 25 nmol/L of BAY11-7082 resulted in a significant decrease in CFU (about 2-fold) compared to the vehicle control group. This effect was more pronounced at concentrations of 50–400 nmol/L (**Fig. 1D**). The discrepancy of CFU to MTT and Annexin-V in the 25 and 50 nmol/L groups suggested that BAY11-7082 might selectively kill colonogenoic cells. A parallel study was also performed using normal HSPCs. A significant growth repression in normal HSPCs by BAY11-7082 was only observed until 400 nmol/L of BAY11-7082 added, as demonstrated by decreased MTT, increased Annexin-V^+^ cells, and reduced CFU (**Fig. 1E–G**). It was known that leukemic cells are a heterogeneous population of cells. The majority of leukemic cells are partially differentiated leukemic blasts (LBs) which lose colony-forming capacity; only a small proportion of leukemic cells are leukemia stem/progenitor cells (LSPCs) which can form colonies (20–25% of Jurkat cells, **Fig. 1D**). Our results show that a low-dose (<400 nM/L) of BAY11-7082 was sufficient to inhibit the CFU ability in Jurkat cells, suggesting that LSPCs are highly sensitive to NF-κB inhibitor treatment. In contrast, normal HSPCs are relatively tolerant to such lower-dose treatment. Interestingly, LBs are resistant to at least a 7–8 times higher dose of BAY11-7082 than are LSPCs.

### NF-κB inhibitor induces JNK activation, and TNF-stimulated JNK activation is independent of NF-κB signaling

**Figure 2:**
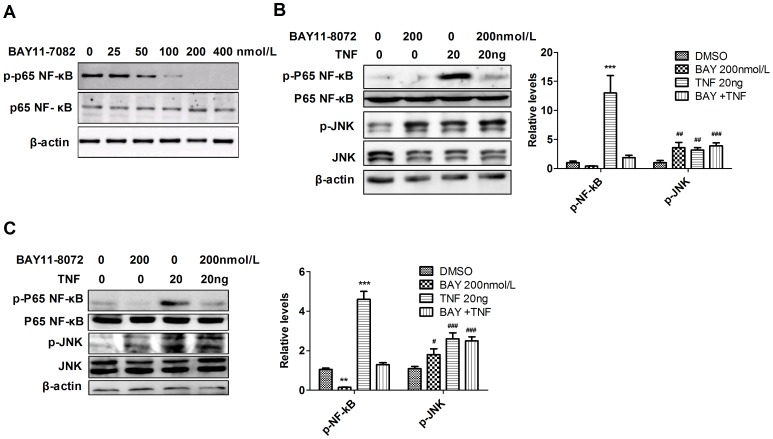
NF-κB inhibitor induces JNK activation, and TNF-stimulated JNK activity is independent of NF-κB signaling. A. Jurkat cells were treated with indicated concentrations of BAY11-7082 for 2 hours. NF-κB activity was examined by Western blotting to detect p-p65 NF-κB levels. B. Jurkat cells were treated with indicated concentrations of BAY11-7082 or TNF alone, or a combination of both, for 1 hour. NF-κB and JNK activity were examined by Western blotting to detect p-p65 NF-κB and p-JNK levels. Bar graphs indicate quantitative densitometry for Western blot bands for both p-NF-kB and p-JNK. C. Normal HSPCs were treated with indicated concentrations of BAY11-7082 or TNF alone, or a combination of both, for 2 hours. NF-κB and JNK activity were examined by Western blotting to detect p-p65 NF-κB and p-JNK levels. Total p65 NF-κB, JNK and β-actin were used as protein loading controls. Data are presented as the means ± SD (n = 3). *P<0.05; **P<0.01; ***P<0.001 compared to DMSO-treated group. #*P*<0.05; ##*P*<0.01; ###*P*<0.001 compared to DMSO-treated group.

Studies demonstrated that many tissue cells in which NF-κB was inactivated showed increased sensitivity to TNF-induced cell death, resulting in severe tissue damage. Studies using knockout mice showed that, in skin and liver, NF-κB inactivation even caused cancer to develop. All such effects of NF-κB inhibition are associated with TNF-induced increased activation of JNK signaling [16]–[18]. To study whether NF-κB inhibition also increases TNF-JNK activity in leukemic cells and HSPCs, we examined JNK activity after BAY11-7082 treatment. We found that in response to increased concentrations of BAY11-7082, p-p65 levels were repressed, suggesting an effect for the inhibitor. Complete suppression of p-p65 was detected at 200 nmol/L of BAY11-7082 (**Fig. 2A**). As expected, we found that expression of p-JNK was increased in both Jurkat cells and normal HSPCs in response to TNF and/or BAY11-7082 treatment. In addition, NF-κB inhibition did not repress TNF- stimulated JNK signaling. These data suggest that: 1) inactivation of NF-κB may induce JNK activation; and 2) TNF-stimulated JNK activation is independent of NF-κB signaling (**Fig. 2B, C**).

### JNK inhibitor SP600125 inhibits the growth of Jurkat cells but has little effect on normal HSPCs

**Figure 3:**
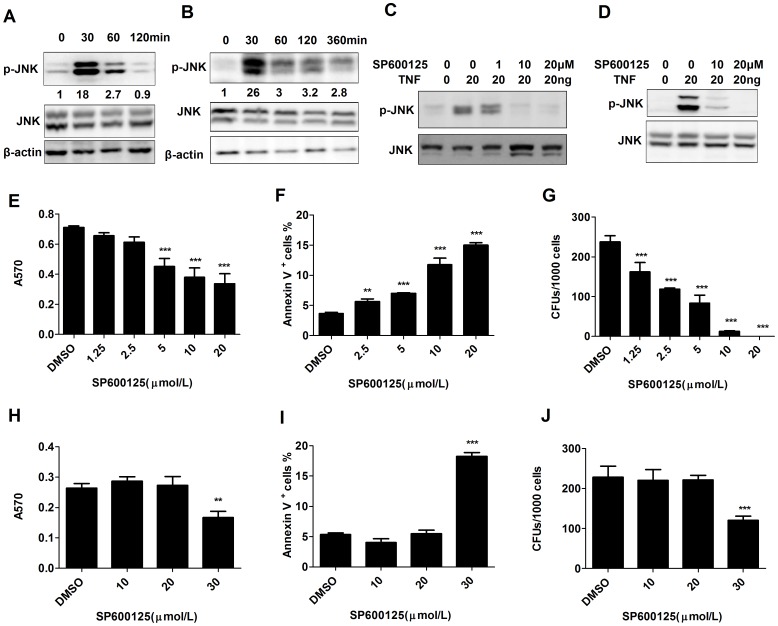
The effect of JNK inhibitor on viability and CFU capacity of Jurkat cells and normal HSPCs. A–B: Jurkat cells (A) and CD34^+^ HSPCs (B) were treated with TNF for 2 hours (A) or 3 hours (B). JNK activity was examined by Western blotting to detect p-JNK levels. Total JNK and β-actin were used as protein loading controls. C–D. Jurkat cells (C) and CD34^+^ normal HSPCs (D) were treated with indicated concentrations of SP600125 together with TNF for 1 hour. JNK activity was examined by Western blotting to detect p-JNK levels. Total JNK and β-actin were used as protein loading controls. E–G: Jurkat cells were treated with indicated concentrations of SP600125. Cells were collected after 48 hours of treatment. E. Cell growth was evaluated by MTT assay; F. cell viability was determined by Annexin-V staining and flow cytometric analysis; G. Colony-forming ability was examined by CFU assay. H–J: CD34^+^ normal HSPCs were treated with indicated concentrations of SP600125. Cells were collected after 48 hours of treatment. H. Cell growth was evaluated by MTT assay; I. Cell viability was determined by Annexin-V staining and flow cytometric analysis; J. Colony-forming ability was examined by CFU assay. Data were collected from triplicate experiments and repeated three times. Data shown in Figures are an average of three independent experiments. *P<0.05, **P<0.01, ***P<0.001 compared to DMSO-treated group.

Consistent with the results of our previous studies in AML cells, TNF induced short-duration JNK activity in Jurkat cells but extended JNK activation in HSPCs (**Fig. 3A, B**). The increased JNK activity in both leukemic cells and HSPCs after NF-κB inhibitor treatment prompted us to inquire about the role of JNK signaling in such cells. Firstly, we determined the optimal concentration of the JNK inhibitor SP600125 by treating Jurkat cells and CD34^+^ HSPCs with TNF to induce JNK signaling activation and then treated with increasing concentrations of the inhibitor. p-JNK was activated by TNF in both Jurkat cells and CD34^+^ HSPCs; levels were down-regulated by SP600125 in a dose-dependent fashion. Complete repression of p-JNK was achieved in Jurkat cells and CD34^+^ HSPCs at an SP600125 concentration of 20 µmol/L (**Fig. 3C, D**). Consistent with these data, dosage-dependent inhibitory effects of SP600125 on the growth and survival of Jurkat cells were observed. We found that 10 µmol/L and 20 µmol/L SP600125 resulted in significant inhibitory effects on the growth and survival of Jurkat cells. After 48 hours of incubation, A570 values of 0.38±0.04 and 0.33±0.03 were obtained for the 10 µmol/L and 20 µmol/L SP600125 treatment groups respectively; these values are significantly reduced compared to the vehicle control group (0.71±0.007) (**Fig. 3E**). Consistently, cell death in the 10 µmol/L and 20 µmol/L SP600125 treatment groups was significantly increased (18.6±1.53% and 22.7±0.58%, respectively) compared to the vehicle control group (3.6±0.21%) (**Fig. 3F**). Furthermore, greater than 50% reduction in Jurkat cell CFUs was observed with an SP600125 concentration of as low as 2.5 µmol/L, and no CFUs were detected when 20 µmol/L SP600125 was used (**Fig. 3G**). In contrast, such dosages of SP600125 had no effect on the survival, growth or CFU ability of CD34^+^ normal HSPCs. The reduction of CFUs for CD34^+^ HSPCs at 30 µmol/L SP600125 might be due to a non-specific activity of this inhibitor (**Fig. 3H–J**) because a complete inhibition of JNK signaling can be achieved at 20 µmol/L SP600125 (**Fig. 3C, D**). Collectively, these data suggest that clonogenic LSPCs are highly sensitive to SP600125 treatment compared to partially-differentiated leukemic blasts (LBs), while normal HSPCs are relatively tolerant to treatment with this inhibitor.

### JNK inhibitor enhances the repressive effect of NF-κB inhibitor on Jurkat cells by promoting extrinsic apoptosis

**Figure 4:**
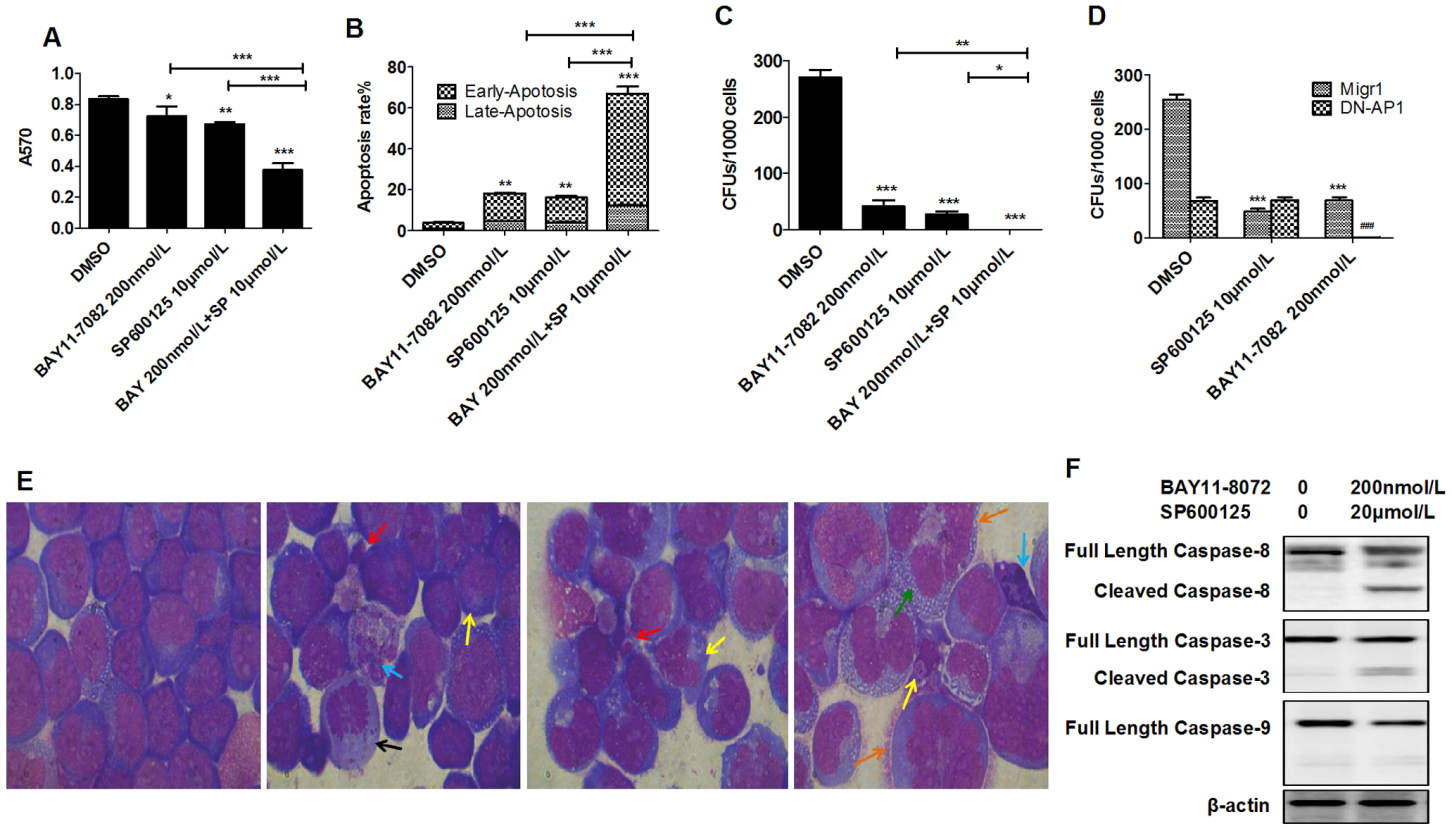
Synergistic effect of NF-κB inhibitor and JNK inhibitor combination treatment on viability and CFU capacity of Jurkat cells. A–C. Jurkat cells were treated with 200 nmol/L BAY11-7082 or 10 µmol/L SP600125 alone, or in a combination using both. Cells were collected after 48 hours of treatment. A. Cell growth was evaluated by MTT assay; B. Cell viability was determined by Annexin-V/PI staining and flow cytometric analysis; C. Colony-forming ability was examined by CFU assay. D. Jurkat cells were transduced with DN-AP1 by viral infection. Transduced cells were purified by FACS and treated with indicated inhibitors or vehicle. Cells were collected after 48 hours for CFU assay. Migr1-vector only transduction was studied in parallel as a control. E. Cell morphology was observed by Wright-Giemsa staining (200×). Black arrows indicate pyknosis; red indicate apoptotic bodies; blue indicate karyorrhexis; yellow indicate nuclear side shift; orange indicate segmented nuclei; green indicate vacuoles. F. The activities of Caspases 8, 9 and 3 were examined by Western blotting to detect the cleaved forms of Caspases. Data are presented as the means ± SD (n = 3). *P<0.05; **P<0.01; ***P<0.001 compared to DMSO-treated group. #*P*<0.05; ##*P*<0.01; ###*P*<0.001 compared to DMSO-treated group.

The findings detailed above imply that both JNK and NF-κB signals are required for the proliferation and survival of leukemic cells, especially LSPCs. Thus the inactivation of both JNK and NF-κB signaling might act in a synergistic manner to kill leukemic cells. To test this hypothesis, Jurkat cells were treated with 200 nmol/L BAY11-7082, 10 µmol/L SP600125 or both of these together in combination for 48 hours. Cell growth and death analysis revealed that the combination treatment yielded a more significant inhibitory/killing effect on leukemic cells compared to individual inhibitor treatment groups. The MTT absorbance in the combination group was 0.38±0.02, significantly lower than either BAY11-7082 or SP600125 individual inhibitor groups, which were 0.72±0.04 and 0.67±0.008, respectively (**Fig. 4A**). The percentages of apoptotic cells were 17.82±0.42% and 16.12±0.92%, respectively, in 200 nmol/L BAY11-7082 and 10 µmol/L SP600125-treated groups, values which are significantly higher than the vehicle control group (3.88±0.18%). The percentage of apoptotic cells in the combined inhibitor group was 66.91±3.11%, which is significantly higher compared to the individual inhibitor-treated groups (**Fig. 4B**). Furthermore, the combination of these inhibitors completely inhibited the formation of colonies whereas a few colonies can still be detected in individual inhibitor treatment groups (**Fig. 4C**). To verify that the inhibitory effects of SP600125 on T-ALL cells are due to the specific inhibition of JNK signaling, Jurkat cells were transduced with DN-AP1 to repress JNK signaling by antagonizing AP1-mediated transcription downstream of JNK. The transduced cells were treated with DMSO, 10 µmol/L SP600125 or 200 nmol/L BAY11-7082 for CFU assay. Migr1-vector-transduced cells were studied in parallel as controls. A significant reduction of CFU was observed in DN-AP1-transduced cells compared to Migr1-transduced cells. Consistent with SP600125 treatment, DN-AP1-transduced cells are more sensitive to BAY11-7082 treatment compared to *Migr1*-transduced cells. Ten µmol/L SP600125 significantly repressed the CFU of Migr1-transduced cells but not DN-AP1-transduced cells, suggesting the JNK-specific effects of SP600125 at dosages used in our experiments (**Fig. 4D**). Collectively, these results indicate that a combination of JNK inhibitor and NF-κB inhibitor results in a synergistic inhibitory effect on LSPC growth/survival by inducing increased apoptosis.

The apoptotic death of Jurkat cells was further verified by cell morphologic study. We found that, after 48 hours of incubation with the indicated concentrations of NF-κB inhibitor or JNK inhibitor, compared to the vehicle control group, the number of apoptotic cells, characterized by segmented nuclei, nuclear side shift, pycnosis, karyorrhexis and apoptotic bodies was significantly increased in both of the individual inhibitor-treated groups; (**Fig. 4E**). We determined that combined NF-κB and JNK inhibitor treatment induced the activation of Caspases 8 and 3 but not Caspase 9. These data demonstrate that JNK inhibition synergizes with NF-κB inhibition to restrict the growth of Jurkat cells by inducing extrinsic apoptotic signaling (**Fig. 4F**).

### JNK inhibitor protects normal human HSPCs from repression by NF-κB inhibitor

**Figure 5:**
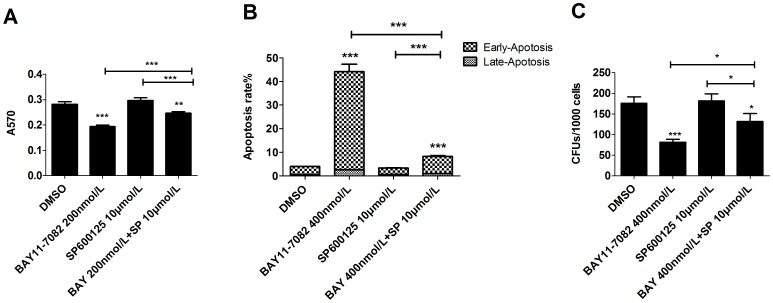
JNK inhibitor protects normal HSPCs from NF-κB-induced apoptosis. A–C. CD34^+^ normal HSPCs were treated with 400 nmol/L BAY11-7082 or 10 µmol/L SP600125 alone, or in a combination using both. Cells were collected after 48 hours of treatment. A. Cell growth was evaluated by MTT assay; B. Cell viability was determined by Annexin-V/PI staining and flow cytometric analysis; C. Colony-forming ability was examined by CFU assay. Data were collected from triplicate experiments and repeated three times. Data shown in Figures are an average of three independent experiments. *P<0.05, **P<0.01, ***P<0.001 compared to DMSO-treated group.

As shown is **Fig. 1E–G**, 400 nmol/L of NF-κB inhibitor significantly repressed the growth, induced death and inhibited CFUs in healthy human HSPCs. We have reported that JNK inhibition prevents normal murine HSPCs from NF-kB inhibitor- induced cell death. To study whether JNK inhibition also protects healthy human HSPCs from the inhibitory effects NF-κB inhibitor, human CD34^+^ HSPCs were treated with 400 nmol/L of BAY11-7082 or 10 umol/L of SP600125 individually or in a combination including both. Cell growth, cell death and CFU were examined after 48 hours of treatment. While 400 nmol/L of NF-κB inhibitor significantly repressed the growth, induced death and inhibited CFUs in HSPCs, we found that such effects were largely prevented by treatment with 10 µmol/L of JNK inhibitor (**Fig. 5A–C**). These results indicate that JNK inhibition also protects normal human HSPCs from the repressive effects of NF-κB inhibition.

## Discussion

Elevated NF-κB activity has often been detected in a variety of types of human malignancy, especially in gliomas, prostate cancer, inflammatory intestinal tumors and leukemias/lymphomas. In these instances, activated NF-κB signaling has been conjectured to contribute to tumor development and progression by promoting the proliferation and survival of tumor cells [28]–[31]. Thus, it was suggested that inhibition of NF-κB signaling is a potentially effective treatment option for these malignancies, inducing longer disease remission and improved patient quality of life and survival [32]. However, the therapeutic use of NF-κB inhibitors can only be evaluated when: 1) the critical role of NF-κB activation in tumor cell growth is firmly confirmed by pre-clinical evidence; and 2) the side-effects of the inhibitors can be minimized.

The role of NF-κB activation in the pathogenesis of hematopoietic malignancies is better suited for study. In B-lymphocytic malignancies, activating mutations of positive regulators or inactivating mutations of negative regulators of NF-κB signaling have been reported, specifically in myeloma, activated B-cell-like diffuse large B-cell lymphoma (ABC-DLBCL), and mucosa-associated lymphoid tissue (MALT) lymphoma, all of which result in constitutive NF-κB activation [33], [34]. These malignant tumor cells are addicted to NF-κB signaling for their proliferation and survival. Thus, NF-κB inhibitors have been evaluated in the treatment of B-ALL/lymphomas, and they showed very promising effects. However, in AML and T-ALL/lymphomas, despite the increased activation of NF-κB in such tumor cells, particularly in LSPCs, mutations of NF-κB signaling components have rarely been reported [35]. The increased NF-κB activity in such tumor cells is induced primarily by: 1) other oncogenic mutations, such as activating mutations; 2) microenvironmental factors secreted by tumor cells or tumor stromal cells; and/or 3) chemotherapeutic-induced DNA damage [36], [37]. Thus, inhibition of NF-κB signaling alone might not be sufficient to eliminate most leukemic cells because other survival/proliferative signaling might be also activated by these oncogenic mutations and microenvironmental factors. Our recent studies with AML cells suggested that TNF-JNK signaling is one such signaling pathway which promotes the survival/proliferation of leukemic cells when NF-κB signaling is repressed. We demonstrated that TNF secreted by AML cells stimulates the survival/proliferation of tumor cells by inducing the activation of NF-κB and JNK, two parallel survival/proliferation signaling pathways. In addition, TNF represses normal hematopoiesis in mice by inducing the activation of JNK-mediated death signaling. Such TNF-JNK death signaling induces tissue damage, which might be the major causes of the side-effects of NF-κB inhibitor treatment. We concluded that inhibition of both NF-κB and JNK signaling should provide a more promising treatment strategy for TNF-expressing AML by synergistically killing leukemic cells while simultaneously providing protection to normal HSPCs [27].

In the current study, we extended our approach to test whether JNK also stimulates NF-κB-independent survival/proliferation signaling in T-ALL cells. Consistent with the conclusions we drew from our studies of AML [37], we found that NF-κB signaling is activated in Jurkat T-ALL cells. Compared to normal HSPCs, LSPCs in Jurkat cells are more highly sensitive to NF-κB-induced cell death. However, inactivation of NF-κB signaling induces the activation of JNK signaling which compromises the killing effects of NF-κB inhibition in Jurkat cells and promote apoptosis in normal HSPCs. Fortunately, inactivation of JNK signaling induces apoptosis in T-ALL cells with negligible influence on normal HSPCs at the optimal concentration of JNK inhibitor, providing a dosage window for us to use JNK inhibitors in the treatment of T-ALL. When used together with an NF-κB inhibitor, the JNK inhibitor amplifies the lethal effects on tumor cells by inducing extrinsic apoptotic signaling while at the same time protecting HSPCs from JNK-mediated cell death.

It is necessary to mention that several previous studies suggested that Jurkat cells are relatively resistant to NF-κB inhibitor treatment. Only minimal apoptosis and growth repression were observed (significance not shown) when 2–3 µmol/L of BAY11-8072 was used [38], [39]. Consistent with these studies, we found that BAY11-7082 induces apoptosis in <20% (4 to 5 times vehicle control) of Jurkat cells even at a concentration as high as 2–3 µmol/L. We believe that such subtle changes can be detected by more sensitive assays and carefully comparisons. Our study demonstrated that a growth repressive effect of BAY11-8072 on CFU in Jurkat cells can be detected at as low as 25 nmol/L and maximal repression can be achieved at 400 nmol/L BAY11-8072. Given a 20–25% frequency of LSPCs in Jurkat cells, as shown by CFU assay, we speculate that BAY11-8072 selectively eliminates LSPCs with limited effects on non-clonogenic LBs. Most importantly, we found that combination treatment using both BAY11-8072 and SP600125 together can completely eliminate LSPCs at a dosage that does not detectably influence the clonogenic capacity of normal HSPCs. Because LSPCs are the cause of leukemia relapse and drug resistance, effective elimination of LSPCs is a very important aspect of successful anti-leukemic treatment. In summary, our findings provide insight into the anti-T-ALL leukemic effect of NF-κB and JNK combined-inhibitor treatment and support further evaluation of these molecules as promising agents for novel therapeutic interventions with other chemotherapeutic agents for the treatment of such fatal diseases as T-ALL.
